# Evaluating Community-Based Intrathecal Baclofen Therapy: Effectiveness, Safety, and Feasibility

**DOI:** 10.3390/jcm13071840

**Published:** 2024-03-22

**Authors:** Simone M. E. van der Gaag, Sander P. G. Frankema, Eva S. van der Ploeg, Sara J. Baart, Frank J. M. P. Huygen

**Affiliations:** 1 Ambulatory Care Clinic, Care4homecare, Rond Deel 12, 5531 AH Bladel, The Netherlands; eva@soulfulbrain.com; 2 Center for Pain Medicine, Erasmus University Medical Center, Doctor Molewaterplein 40, 3015 GD Rotterdam, The Netherlands; s.frankema@erasmusmc.nl (S.P.G.F.); s.baart@erasmusmc.nl (S.J.B.); f.huygen@erasmusmc.nl (F.J.M.P.H.)

**Keywords:** spasticity, neuromodulation, ambulatory care, intrathecal baclofen, screening, ITB trial, aftercare

## Abstract

**Background:** Intrathecal baclofen (ITB) is used for the treatment of intractable spasticity. The burden of traveling for ITB screening and aftercare is problematic for nursing home residents with severe spasticity and seems to result in undertreatment of spasticity. The aim of this study is to evaluate the effectiveness, safety, and feasibility of ITB for nursing home residents treated in their home, describing the selection phase, the initial trial of ITB, and aftercare up to 3 months after implantation of an ITB pump. **Methods**: This retrospective database study included immobile, adult nursing home residents with severe spasticity, referred to an Ambulatory Care Clinic between 2016 and 2021. When eligible, an ITB trial was performed by ITB experts in the nursing home. If a permanent pump was implanted, dose titration and aftercare were performed on location. **Results**: A total of 102 patients were referred; 80 underwent an ITB trial on location, and 94% improved significantly on the Modified Ashworth Scale and clonus scale pre-ITB trial versus post-ITB trial, as well as at 3 months post-implantation. There was a low incidence of adverse events, mostly procedure- and drug-related. **Conclusions**: This study indicates that selection, testing, and aftercare for ITB on location is effective and safe.

## 1. Introduction

Spasticity is a common symptom in central nervous disorders such as stroke (28%–40%), traumatic brain injuries (BIs) (13%), spinal cord injuries (70%), cerebral palsy (CP) (90%), and multiple sclerosis (MS) (41–80%) [1,2,3,4,5,6]. Amongst people who reside in nursing homes (NHs) with these pathologies, the prevalence of spasticity is 73% [7]. Spasticity symptoms vary from subtle neurological symptoms to a gross increase in muscle tone causing immobility of joints, contractures, involuntary movements, and pain. Severe spasticity can have a detrimental effect on activities of daily living, sleep patterns, and overall quality of life, consequently resulting in a need for nursing care and thus care dependency [8].

In most cases where spasticity is disabling, pharmacological treatment with spasmolytics is offered. Baclofen is a well-known and widely used option, but its oral application has, at least in some patients, many side effects such as sedation, concentration problems, and memory deficits, particularly when used in higher dosages. Direct administration of baclofen into the cerebrospinal fluid (intrathecal baclofen (ITB)) significantly reduces severe spasticity, whereas the side effects associated with oral intake are mostly eliminated [9,10,11,12,13,14].

In the Netherlands, ITB for severe spasticity has been an accepted treatment for decades for patients whose oral treatment has insufficient effect and/or too many side effects. ITB is available worldwide. However, it seems that ITB is not a common treatment option and is often seen as a last resort. Meijer et al. stated that there is a high prevalence of spasticity, with a substantial impact on caregiving and complaints, in nursing home patients with central nervous system disorders [7]. According to Erwin et al., who focused particularly on patients with MS, this undertreatment begins with an underestimation of the impact of spasticity on quality of life. Erwin et al. concluded that ITB was underutilized due to a focus on disease-modifying therapies rather than symptom management [15]. In addition, undertreatment of severe spasticity is more common in nursing home residents, ref. [15] which may be explained by the burden of commuting to the hospital for numerous visits for referral, selection, testing, preoperative anesthesiology assessment, implantation, and aftercare [16]. To decrease this burden of commuting on patients, a nationwide Ambulatory Care Clinic was founded in 2010 in the Netherlands. The Ambulatory Care Clinic performs ITB procedures on location where the patient resides [17]. When a nursing home resident is deemed eligible for ITB treatment, the Ambulatory Care Clinic can be consulted for a visit at the patient’s home for an intake and the ITB trial that precede the possible implantation of a permanent pump. After pump installation, the Ambulatory Care Clinic also performs ITB aftercare, including pump refills, again at the place where the person resides, not in the hospital. Although several studies have proven the effectiveness of ITB to reduce spasticity, this new application of ITB in an outpatient setting for people who need it most (both in terms of the prevalence of severe spasticity as well as the lack of appropriate treatment) has not yet been extensively studied [15]. ITB treatment in an ambulatory setting for nursing home residents adds an important dimension to the quality of life for this patient group. The aim of this study is to evaluate the effectiveness, safety, and feasibility of ITB for nursing home residents treated in their home, describing the selection phase, the initial trial of ITB, and aftercare up to 3 months after implantation of a permanent ITB pump.

## 2. Materials and Methods

This retrospective database study of prospectively collected data was conducted in accordance with the Declaration of Helsinki and approved by the Institutional Review Board (Ethics Committee) of Erasmus Medical Center (protocol code: MEC-2022-0096 and date of approval: 21 February 2022). After referral to the Ambulatory Care Clinic, patients gave consent for treatment at the initial visit. Patient consent was waived, which is common in database studies. This study includes patients with severe disabling spasticity due to a variety of central nervous disorders referred between 2016 and 2021 to Care4homecare, the Ambulatory Care Clinic with headquarters based in the south of the Netherlands and nationwide reach of outpatient care.

Referral to the Ambulatory Care Clinic: The Ambulatory Care Clinic enrolled immobile, adult residents from different NHs and disability communities suffering from severely disabling spasticity caused by a variety of etiologies. An additional inclusion criterion for an ITB trial was that patients had not experienced significant spasticity relief from previous or ongoing, more conservative treatments. To check the success of previous treatments, the Ambulatory Care Clinic requested the recorded patient history. An anesthesiologist–pain physician specialized in ITB reviewed the written information (including comorbidity) and performed a patient evaluation on location. During this process, the Ambulatory Care Clinic provided detailed information to patients, family, and caregivers about the advantages and disadvantages of ITB treatment.

Then, in a multidisciplinary meeting with the NH physician and the rehabilitation physician, the Ambulatory Care Clinic discussed the case and treatment goals. For immobile patients, the treatment goals are for symptomatic relief of pain and spasm to make nursing care and sitting easier [15]. When warranted, the discussion involved a neurologist, neurosurgeon, internist, neuroradiologist, psychologist, and/or physiotherapist. If the patient was selected as a potential candidate and they consented to an ITB trial, the next phase of the process was initiated, namely preparation for a trial of ITB. ITB candidates were given at least a week to process the information and make a final decision to participate in the ITB trial.

An anesthesiologist–pain physician and nurse practitioner (NP) (both ITB specialists) performed the ITB trial in the NH or disability community. During the ITB trial, the physician and NP would stay in the NH for at least three hours after the trial administration of ITB. After they had left, the NH physician, physiotherapist, and nurse observed the patient throughout the day, including transfers from bed to wheelchair, sitting in the wheelchair, and assistance with dressing and undressing. This information was shared with the Ambulatory Care Clinic.

To assess a patient’s response to ITB, a single bolus injection (50 micrograms [mcg]) was administered via a lumbar puncture (LP) during the trial [18]. If needed, we prolonged the ITB trial period by one or two days for a second or third injection, with a maximum dose of 100 mcg. Patients were again monitored in a similar manner as after the first gift. During the ITB trial, we made no change to any oral spasmolytic medication. In cases where patients were receiving anticoagulant or antiplatelet therapy, we discontinued this treatment, as indicated by the guideline for locoregional anesthesia and anticoagulation of the Dutch Association of Anesthesiology [19]. During the ITB trial, the Ambulatory Care Clinic brought all necessary equipment, including trial medication, monitoring equipment, and an emergency kit to intravenously administer atropine, clemastine, and ephedrine. The Ambulatory Care Clinic shared a detailed written consultation letter after each visit, which included 24 h emergency contact information.

Measurements of vital signs (blood pressure, heart rate) before, immediately after, and 1 and 2 h after the lumbar puncture were performed. Before and two hours after intrathecal injection of baclofen, we assessed the severity of spasticity in all four extremities using the Modified Ashworth Scale (MAS) and clonus scale [20]. We assessed wrist and elbow flexors and extensors as well as ankle dorsiflexion and plantar flexion, knee flexion and extension, and hip adduction and abduction [20]. The MAS ranges from 0 (no increase in tone) to 4 (extreme rigidity) [21]. The clonus scale is derived from the Tardieu scale, with 1 signifying fatigable clonus (<10 s (seconds)) and 2 indefatigable clonus (>10 s) [21]. (Serious) adverse events ((S)AEs) were recorded. We defined an AE as any unfavorable medical occurrence causing discomfort that was self-limiting or that could easily be treated. In a moderate or severe event, hospital admission was indicated. SAEs were events that caused death, were life-threatening, or caused disability or permanent damage to the patient [22].

When the ITB trial resulted in at least a two-point reduction in the total scores of the spasticity and clonus scales, and the patient, the patient’s family, and the treatment team also clinically confirmed the reduction in spasticity, we regarded the patient as a suitable candidate for a permanent ITB pump. We offered patients or their legal representatives one week of reflection before they decided to get a permanent ITB pump installed. On confirmation, we referred patients to a neuromodulation center for implantation of the pump (SynchroMed II^®^, Medtronic Inc., Minneapolis, MN, USA). After implantation, patients stayed one night in the hospital before being discharged to their NH or disability community. Usually, the starting daily dose would be double the single bolus dose required to attain a recognizable spasmolytic effect. The NP performed dose titration twice a week with a maximum daily dose increment of 10%. After the Ambulatory Care Clinic staff observed the desired decrease in spasticity, the correct delivery amount was established and maintained. Any oral spasmolytic medication was discontinued. Following the initial dose titration phase, patients were included in the continuous aftercare program of the Ambulatory Care Clinic, in which NPs perform dose adjustments, program the pump, and refill procedures on location [17].

### Statistical Analysis

Continuous outcomes are presented as median [interquartile range] and categorical outcomes as counts (percentages). Differences in continuous outcomes on the time points were compared using Wilcoxon signed-rank tests to account for the paired nature of the observations. Differences in binary outcomes between the time points were compared with the McNemar test. Two-sided *p*-values of < 0.05 were considered statistically significant. The statistical analyses were performed with R version 4.1.0.

## 3. Results

### 3.1. Demography

In the period between 2016 and 2021, a total of 102 patients were referred to the Ambulatory Care Clinic, of whom 76 (77.2%) were living in NHs and the others in disability communities (Table 1). The sample contained more males than females. The median age was 51. One-third had a diagnosis of MS, and around 20% had CP or BI.

### 3.2. Outcomes

#### Feasibility

**Figure 1:**
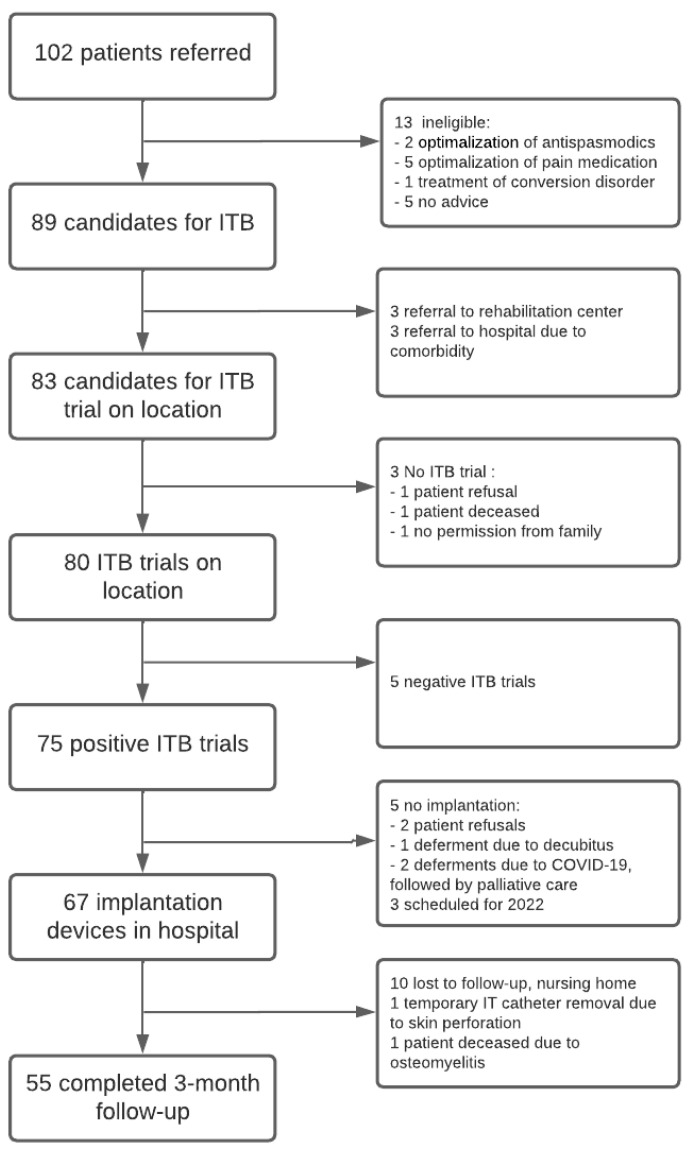
Flow diagram of patients suitable for the ITB trial and, after a positive result, for referral for implantation and aftercare.

Of the 102 patients referred, 89 met the inclusion criteria for an ITB trial (Figure 1). The ITB trial group looked similar to the complete sample (Table 1). Of the 89 patients, 3 were referred to the rehabilitation center, for they had high-level goals such as walking and standing, and 3 were referred for an in-hospital trial due to their comorbidity. We considered 83 suitable for an ITB trial on location, for they had passive goals, such as improved positioning and wheelchair tolerance, improved daily care (decreased caregiver burden and time), and improved quality of sleep, and had tried two or more oral spasmodic agents in the past. Of these, one patient refused the ITB therapy, one patient died whilst on the waiting list, and one patient’s family did not give informed consent for the ITB trial. In summary, 80 patients underwent an ITB trial on location. 

**Figure 2:**
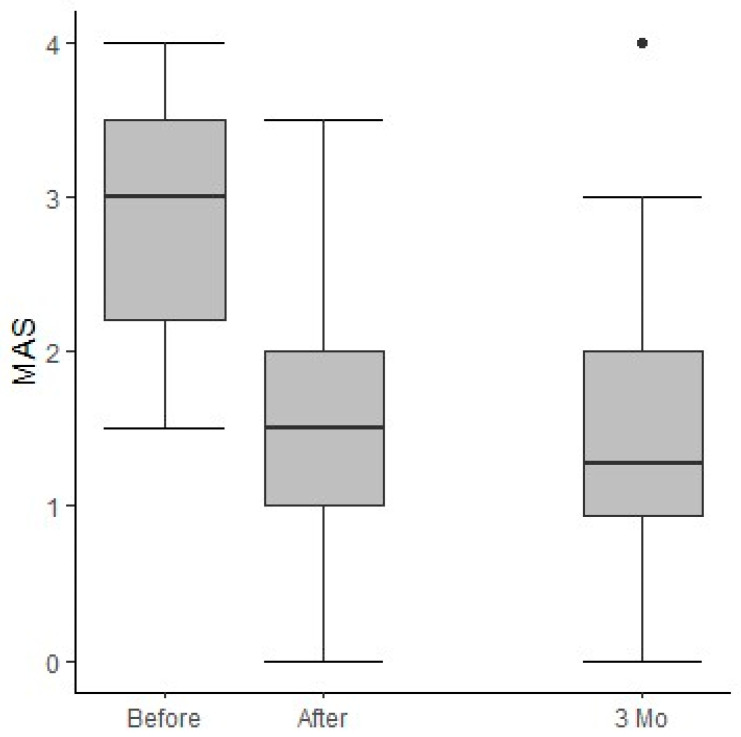
Median and IQR of MAS scores pre-ITB trial, two hours after the ITB trial, and 3 months after the implantation of a permanent pump (*n* = 80).

Of the 80 screened patients, 75 (93.8%) showed a relevant improvement in their spasm scores following the trial of ITB. The measured MAS scores pre-ITB trial (median: 3 [interquartile range (IQR): 2.2–3.5]) vs. two hours post-ITB trial (median: 1.5 [IQR: 1.0–2.0]) decreased significantly (*p*-value: <0.0001). Not all patients showed intentional or spontaneous spasms and/or clonus before treatment. If present, the measured clonus scale score decreased significantly (*p*-value: <0.0001). Three months post-implantation of the ITB device, the MAS (median: 1.3 [0.9–2.0]) (*p*-value: <0.0001) and clonus scores (*p*-value: <0.001) were still significant compared to the pre-ITB trial scores. Comparing the 3-month data with the post-implantation data, the MAS (*p*-value: 0.7189) and clonus (*p*-value: 0.3864762) scores did not decrease significantly (Figure 2). Supplementary Material Figure S1 is providing detailed information on MAS. 

During the ITB trial, five patients did not respond to the maximum dose of baclofen (100 mcg). For these five (6.3%), we considered the test to be unsuccessful. We made the decision for permanent implantation after one bolus of 50 mcg baclofen in 65 patients (81%), after two injections (75 mcg) in 3 patients (4%), and after three injections (100 mcg) in 7 patients (9%). Ultimately, of the 75 patients with a successful ITB trial, 67 received a permanent device implant (Figure 1). Two patients refused an implantation, three patients died (one due to osteomyelitis and two due to COVID-19) whilst on the waiting list, and three patients were scheduled for 2022 and therefore outside the study population.

Most of the patients (80%) used oral antispasmodics in the pre-surgical treatment phase: baclofen (52/80), tizanidine (18/80), tolperisone (15/80), dantrolene (6/80), and clonazepam (6/80). During the ITB trial, the oral antispasmodics were continued. Because abrupt discontinuation of oral antispasmodics can cause withdrawal and the effects of ITB therapy are somewhat delayed after permanent treatment starts, oral antispasmodics were tapered off in steps [21]. Three months post-implantation, six patients (7.5%) still used (a lower dose of) oral baclofen.

### 3.3. Treatment Goals

Together with the patients (*n* = 80) or their representatives, professionals formulated one or several treatment goals. The goals included reducing spasms (80/80), making nursing care easier (78/80), improving sitting position (30/80), reducing pain (26/80), improving lying position (10/80), improving sleep (8/80), reducing drowsiness (4/80), improving passive transfer (4/80), and preventing contractures (2/80). Preventing contractures is a long-term goal and cannot be tested during a short ITB trial. And since oral antispasmodics were continued during the ITB trial, the effect on drowsiness could only be assessed after implantation and discontinuation of the oral medication. The five patients who did not experience reductions in spasms during the ITB trial also did not report a reduction in pain.

### 3.4. Safety

**Figure 3:**
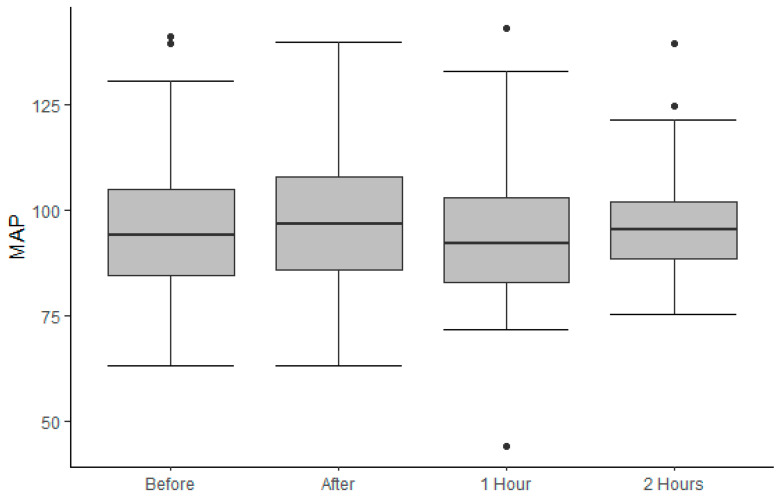
Measurement of MAP. Predefined moments are before and immediately, 1 h, and 2 h after the lumbar puncture.

During the ITB trials, none of the patients was in need of rescue medication. Details are given in Figure 3. In one patient, the measured mean arterial pressure (MAP), one hour post-lumbar puncture, was <60 mm of mercury (mmHG) and spontaneously recovered to 95 mmHG within 30 min. The patient was responsive and experienced no health complaints. Supplementary Material Figure S2 is providing detailed information on MAP.

#### AEs and Severe AEs

Throughout the study, we recorded (S)AEs (Table 2) during the ITB trial, after implantation of the pump device, and at three-month follow-up. During the ITB trial, four patients (5%) suffered from an AE (one had post-dural puncture headache (PDPH), one had radiating pain, one had a cotton-ball feeling in the head, and one had nausea). In 26 patients (38.8%), a post-implantation AE occurred. Most of the AEs were procedure- and drug-related. Between implantation and three-month follow-up, eight patients (14.5%) had an AE. In two patients, there was no decrease in spasticity, and they were referred to the neuromodulation center for diagnostic evaluation. Both patients needed revision of the spinal catheter.

## 4. Discussion

This study summarizes seven years of clinical experience with the selection, testing, and aftercare of intrathecal baclofen therapy in an outpatient setting for patients with severely disabling spasticity (*n* = 102). Of the nursing home (or disability community) residents who were enrolled in an ITB trial, 93.8% showed a relevant improvement in their spasm scores and thus were identified as eligible for implantation of a permanent ITB delivery system. During the titration phase post-implantation, the MAS score decreased significantly. The difference in MAS scores between pre-ITB trial and three months post-implantation decreased significantly. Comparing the 3-month data with the post-implantation data, the MAS (*p*-value: 0.7189) and clonus (*p*-value: 0.3864762) scores did not further decrease nor did they return to baseline. In other words, the improvements lasted over time. These results are similar to those of ITB care in an inpatient setting [23].

The most commonly used oral antispasmodic in the pre-surgical treatment phase was baclofen. Most patients were able to reduce and stop oral antispasmodics within three months after pump implantation. Goals for this immobile group of patients were formulated by patients themselves or their representatives. The effect of the ITB trial on their goals was asked or observed.

Four AEs and no SAEs occurred when the ITB trials were performed by the Ambulatory Care Clinic. During the post-operative phase, 32 (S)AEs occurred, and in the follow-up phase 8 (S)AEs occurred. Compared to the literature, there was a low incidence of (S)AEs [24]. Therefore, we can conclude that ITB care provided by an Ambulatory Care Clinic does not entail any additional risk. (S)AEs will always occur with this complex/intrusive procedure, regardless of the setting. It is therefore important to prevent (S)AEs as much as possible, but also to recognize the indicators if they do occur. As Saulino et al. described, symptoms of complications should be recognized and treated by an ITB-experienced team [24].

We realize there are limitations to this study. We collected the data retrospectively, the data were sometimes incomplete, and the data were based on a relatively small study sample. We did not make a controlled randomized comparison between in- and outpatient ITB pathways. We have no knowledge of other Ambulatory Care Clinic data for comparative analyses. No questionnaire was used to measure the effect of the test on the objectives. Therefore, we do not have quantitative data. Although long-term goals such as the prevention of contractures could not be measured during the ITB trial, the literature confirms that ITB can prevent contractures [25].

Another limitation is that the ITB team stayed in the NH for three hours after administering the treatment. The team assessed spasticity before treatment and two hours after treatment. A recently published guideline on best practices for ITB therapy advises extending this observation period to four hours, as some patients could be late responders [21]. However, after the ITB team left, the nursing home team continued to observe the patient throughout the day. Due to the fact that there is always the risk of an inadvertent extradural injection or aberrant spread of the medication, we repeated all negative tests with an increased dose of up to 100 mcg. We feel that this limited the number of falsely omitted suitable candidates due to the shorter observation time.

Finally, the Netherlands is a relatively small country, and we realize that home-based ITB delivery might be more challenging to organize in countries with longer travel distances and in more remote areas.

Despite these limitations, we have shown that the selection, testing, and aftercare of intrathecal baclofen therapy in an ambulatory setting for patients with severely disabling spasticity living in a NH is effective, feasible, and safe, with no increase in complications compared to when performed in the hospital.

To be eligible for ITB therapy, patients had to have an estimated life expectancy of at least one year. However, one patient died before the ITB trial was performed and one patient in the three-month follow-up phase due to osteomyelitis. Finally, for two patients who had a positive ITB trial, implantation of the pump device was deferred because they suffered from SARS-CoV-2 infection and were transitioned into palliative care. There was no indication in the patient files that these deaths were related to the ITB therapy.

In retrospect, considering the severe level of suffering of people with severely disabling spasticity, we were surprised by the treatment refusal by patients (*n* = 2) themselves of this likely effective treatment option. This may be related to unfamiliarity with the procedure. Although not the focus of this study, we got the impression that when the therapy was better known in a particular NH or disability community, more patients were referred. Although certain criteria, such as life expectancy, can be established, it is essential to balance this with respect for the patient’s right to choose the course of action they prefer, including refusal of treatment. Patient decisions may evolve over time, and ongoing communication is vital. Regularly checking in with patients, providing additional information, and addressing new concerns stimulate a collaborative approach to decision making. Patients may change their minds given sufficient time and support. In addition, center-wide introductory sessions of ITB treatment could decrease the level of refusal.

In this study, we attempted to implement a comprehensive set of measures to address potential biases, encompassing transparency, stringent patient selection criteria, patient-centered approaches, extended observation periods, considerations of team expertise, acknowledgment of geographical challenges, and ethical considerations regarding patient refusals.

Undertreatment of disabling spasticity is a worldwide phenomenon and is especially prevalent in nursing home settings, where ITB is rarely offered [15]. This undertreatment can at least be partly explained by the complexity of the set-up required to deliver the therapy and the need for interaction between several medical specialists (including neurosurgery, anesthesiology, and neurorehabilitation specialists, all of whom may not be present in one location) [26]. Offering ITB by means of an Ambulatory Care Clinic seems to be one possible solution to overcome these challenges.

To build on the outcomes of this study, future research can focus on prospectively assessing the impact of delivering ITB directly to patients in nursing homes, for example by means of a Randomized Controlled Trial. This could explore how ITB influences their daily functioning and overall quality of life. Understanding the practical implications of home-based ITB therapy is essential for optimizing patient outcomes and potentially addressing the existing gap in spasticity management, especially in the context of nursing home care.

## 5. Conclusions

The results of this study indicate that selection, testing, and aftercare for ITB in an outpatient setting is as effective and safe as in the hospital setting. Ambulatory care prevents the burden, and any other negative impact, of traveling, which is of the utmost importance for immobile patients with severe spasticity and other symptoms such as pain. This study shows that an Ambulatory Care Clinic, in close collaboration with a neuromodulation center, presents an innovative alternative. Despite the relatively small sample and other limitations of this study, we feel these outcomes are an essential starting point to deliver ITB treatment to those most vulnerable and most in need of it. Introducing Ambulatory Care Clinics worldwide could potentially be a crucial step in reducing ITB undertreatment.

## Figures and Tables

**Table 1:** Demographic and baseline characteristics of total sample (*n* = 102) and those eligible for an ITB trial (*n* = 89).

	Referred Population	Eligible for ITB Trial
	***n* = 102**	***n* = 89**
**Gender**		
Sex, male *n* (%)	64 (63.0%)	59 (66.3%)
Age, median (IQR)	51 (39–60)	51 (39–59)
**Care institution**		
Nursing home	76 (77.2%)	
Disability communities	26 (26.5%)	
**Disease**		
Multiple sclerosis	36 (35.3%)	31 (34.8%)
Cerebral palsy	20 (19.6%)	16 (18.0%)
Brain injury	19 (18.6%)	19 (21.3%)
Spinal cord lesion	9 (8.8%)	6 (6.7%)
Stroke	9 (8.8%)	8 (9.0%)
Others	9 (8.8%)	9 (10.0%)

**Table 2:** (S)AEs during ITB trial, post-operation, and within the three months of follow-up.

	ITB Trial	Post-Operation	3-Month Follow-Up
Patient total	*n* = 80	*n* = 67	*n* = 55
Patients with (S)AEs	*n* = 4 (5%)	*n* = 26 (38.8%)	*n* = 8 (14.5%)
Number of complications	4	32	8
Procedure-related	2/4	15/32	3/8
PDPH	1	6	
Constipation		3	
Pocket pain			1
Radiating pain	1		
Hematoma pocket		1	
Perforation of intrathecal catheter skin		1	
Blisters due to band aid		1	
Pressure ulcers		2	
No effect on spasticity			2
Incorrect programming (concentration of baclofen)		1	
Drug-related	2/4	11/32	1/8
Cotton-ball feeling in head	1		
Loss of strength		2	1
Urinary retention		2	
Somnolence		5	
Nausea/emesis	1	2	
Patient-related	0/4	6/32	4/8
Autonomic dysregulation		1	
Urinary catheter blockage			1
Hyperglycemia			1
Hypercalcemia		1	
Urinary tract infection		1	
SARS-CoV-2 infection			2
Pneumonia		2	
Unclarified increased C-reactive protein (CRP) level		1	

## Data Availability

Data are available upon request from the authors.

## Supplementary Materials

The following supporting information can be downloaded at: https://www.mdpi.com/article/10.3390/jcm13071840/s1, Figure S1: Data on MAS. Pre-ITB trial, two hours after intrathecal injection of baclofen, and 3 months after implantation of the pump device; Figure S2: Data on MAP. Predefined moments are before and immediately, 1 h, and 2 h after the lumbar puncture.

## Abbreviations

AE	Adverse event
BI	Traumatic brain injuries
CP	Cerebral palsy
IQR	Interquartile range
ITB	Intrathecal baclofen
LP	Lumbar puncture
MAP	Mean arterial pressure
MAS	Modified Ashworth Scale
Mcg	Micrograms
mmHG	Millimeter of mercury
MS	Multiple sclerosis
NH	Nursing home
NP	Nurse practitioner
PDPH	Post-dural puncture headache
SAE	Serious adverse event
Sec	Seconds
