# Supplementary figures and images for: Evaluating Community-Based Intrathecal Baclofen Therapy: Effectiveness, Safety, and Feasibility
Source: J Clin Med. 2024 Mar 22;13(7):1840. doi: 10.3390/jcm13071840 (PMC11012490; doi:10.3390/jcm13071840)

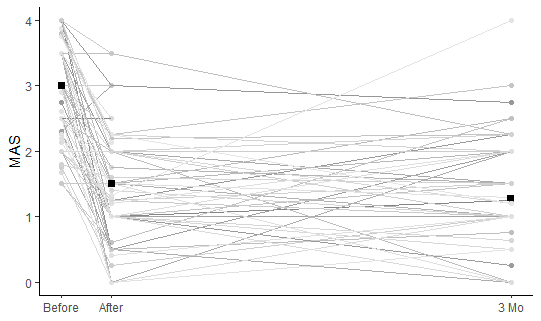

Supplement: Supplementary file 1 [file jcm-13-01840-s001.zip › Figure S1. Data on MAS.png]

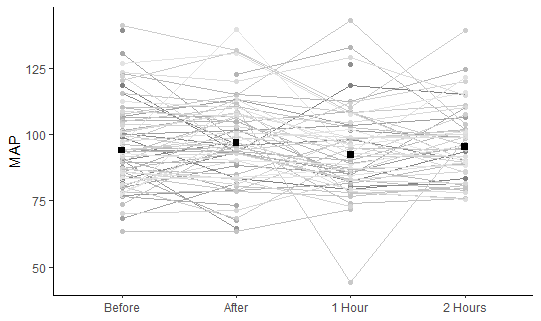

Supplement: Supplementary file 1 [file jcm-13-01840-s001.zip › Figure S2. Data on MAP.png]
